# Uncompatibilized PBAT/PLA Blends: Manufacturability, Miscibility and Properties

**DOI:** 10.3390/ma13214897

**Published:** 2020-10-31

**Authors:** Shen Su, Mona Duhme, Rodion Kopitzky

**Affiliations:** 1 Department of Circular and Bio-based Plastics, Fraunhofer UMSICHT, Institute for Environment, Safety and Energy Technology, Osterfelder Str. 3, 46047 Oberhausen, Germany; mona.duhme@umsicht.fraunhofer.de (M.D.); rodion.kopitzky@umsicht.fraunhofer.de (R.K.); 2 Department of Mechanical Engineering, Ruhr-University Bochum, Universitaetsstr. 150, 44780 Bochum, Germany

**Keywords:** blend, miscibility, morphology, PBAT, tear propagation resistance

## Abstract

Polymer blends of poly(butylene adipate-co-terephthalate) (PBAT) and polylactide (PLA) have been drawn attention due to the application potential as packaging or agricultural films. This study aims to determine the manufacturability, miscibility and mechanical properties of uncompatibilized PBAT/PLA blends prepared using different techniques. First, PBAT and PLA are melt-blended in a wide range of ratios from 90/10 to 10/90. The compounds are then processed into pressed panels, flat films and blown films. Finally, the thermal, morphological, rheological and mechanical properties of these blends are investigated. PBAT/PLA blends have a small difference of solubility parameters, predicting theoretically good miscibility. However, they show two almost unchanged glass transition temperatures in the DSC, phase separation in SEM and two relaxation mechanisms in the Cole–Cole plot. The phase morphology varies depending on both the blend ratios and the preparation techniques. Tensile tests indicate that with increasing PLA content the elongation at break decreases. A good correlation between the elongation at break and the tear propagation resistance is found. Furthermore, the trouser tear method is proven to be more applicable to differentiate highly extensible blown films compared with the Elmendorf tear method.

## 1. Introduction

To achieve sustainable development goals, environmentally friendly polymers have been increasingly developed in recent years. As commercially available biopolymers, poly(butylene adipate-co-terephthalate) (PBAT) and polylactide (PLA) currently account for 7.2 and 10.3 percent of global bioplastics production capacity, respectively [1]. PBAT is an aliphatic-aromatic copolyester [2,3], which is biodegradable, highly flexible and designed for film extrusion [4,5]. However, its low thermo-mechanical properties restrict its utilization [6]. Compared with PBAT, PLA exhibits attractive mechanical and physical properties such as high tensile strength and modulus [4]. Moreover, PLA can be produced from renewable resources and biodegrades under industrial composting conditions [7]. However, PLA suffers from its brittleness and low toughness at ambient conditions [4], due to its molecular structure with a relatively rigid backbone and small methyl side groups.

Melt blending is a method to mix some polymers without chemical reactions taking place [8]. Through melt blending, flexible PBAT and stiff PLA can achieve improved material properties without losing the biodegradability. In recent years, numerous studies have been performed on PBAT/PLA blends, but the dimensions of the test specimens, the production methods and measurement conditions used are different so that the tensile test results are difficult to compare. In a PBAT/PLA blend, PBAT can act either as a dispersed component [9,10] or as a polymer matrix. When PBAT is added into the PLA matrix as a dispersed component, different studies point that the elongation at break increases, but the tensile strength and modulus of elasticity decrease with increasing PBAT content (10–40 wt.%) [9,10,11]. When the PBAT content increases from 40 to 60 wt.%, the elongation at break increases dramatically; the modulus of elasticity and tensile strength decrease slightly [12]. When PBAT acts as a polymer matrix, i.e., PLA is the dispersed component, the elongation at break increases, but the modulus of elasticity decreases with decreasing PLA content [13]. Commercialized products (Ecovio^®^) made of PBAT/PLA blends have been developed by BASF [2]. However, the high cost (≈5 $/kg) and the insufficient material properties still limit the applications [14]. A competitive material cost is achievable by blending PBAT and PLA with a high PLA content, because compared with PBAT, PLA is now relatively inexpensive.

However, the material properties of a polymer blend depend strongly on the processing and the miscibility of the components. To develop polymer blends with desired costs and properties, it is necessary to determine the manufacturability using different production techniques and the blend miscibility in different compositions. In this research, uncompatibilized PBAT/PLA compounds with a wide range of ratios (from 90/10 to 10/90) are melt-blended. Then the blend manufacturability into pressed panels, flat films and blown films is discussed with the blend miscibility, providing an important basis for modifying PBAT/PLA blends and optimizing the processing parameters.

To investigate the phase behavior of binary polymer blends, frequently used methods include differential scanning calorimetry (DSC), scanning electron microscopy (SEM) and rheology. The DSC analysis can indicate whether a polymer blend has only one single glass transition temperature if the blend were fully miscible. SEM shows whether the blend is in a homogeneous state or a phase-separated state. The Cole–Cole-plot of rheology displays whether the blend has two relaxation mechanisms correspond to the phases and whether the phase transition takes place.

Recently, a few studies have been carried out regarding blown films made of PBAT/PLA blends. Pietrosanto et al. found that blown films with the composition of PBAT/PLA (80/20) (thickness: 75 ± 5 µm) have potential as frozen food packaging material [15]. Kim et al. reported that the PBAT/PLA (65/35) blown film (thickness: 30 µm) achieves an elongation at break of approximately 304% and 235% in the machine direction (MD) and cross direction (CD), respectively. Meanwhile, these blown films have a tear propagation resistance of about 4.6 N/mm and 8.7 N/mm in the MD and CD, respectively [16]. In addition to the tensile properties, tear propagation resistance is also required for blown film applications. Tear propagation resistance is commonly determined using the Elmendorf tear test [17,18] or trouser tear test [19,20]. In this study, both test methods are used and compared in terms of their suitability for measuring high extensile samples. The correlation of the tear propagation resistance to other mechanical properties is discussed.

The study mainly aims to explore the manufacturability and miscibility of uncompatibilized PBAT/PLA blends in a wide range of ratios and the influence of the blend ratios on the rheology, morphology and mechanical properties. Furthermore, this study will provide a basis for the PBAT/PLA blend modification and the optimization of processing parameters, especially for blown films. The novelty of this work is primarily the applicability comparison of two tear test methods (Elmendorf and trouser tear) for differentiating PBAT/PLA blown films and determination of the correlation between the tear propagation resistance and the tensile properties.

## 2. Materials and Methods 

### 2.1. Materials

PBAT (Ecoflex F Blend C1200, BASF SE, Ludwigshafen, Germany) exhibits a weight average molecular weight of 1.05 × 10^5^ g/mol and a polydispersity of 2.0.

PLA (Ingeo^TM^ Biopolymer 2003D, NatureWorks LLC, Minnetonka, MN, USA) exhibits a weight average molecular weight of 2.10 × 10^5^ g/mol, a polydispersity of 1.6 and a D-isomer content of approximately 4.4%.

The neat polymer granules are pre-dried at 60 °C for 2 h in a hot air oven before use.

### 2.2. Blend Preparation

The procedure of the blend preparation is described as follows: The pre-dried polymers with different weight ratios (PBAT/PLA: 90/10, 80/20, 70/30, 60/40, 50/50, 40/60, 30/70, 20/80 and 10/90) are premixed and dosed into a gravimetric dosing unit (DDW-H31-FW33/2-17, Brabender Technologie, Duisburg, Germany). Subsequently, the formulations are melt-blended using a co-rotating twin-screw extruder (EMP 26-40, TSA Industriale S.r.l., Luisago, Italy) with a diameter of 26 mm, a length to diameter ratio of 40:1. The screw rotation speed is set at 200 rpm. The temperature profile is set to 150*/*160*/*170*/*170*/*170*/*170*/*170*/*170 °C from the feed zone to the die. After melt blending, each strand is quenched in a water bath and then pelletized. Finally, the pellets are dried at 60 °C.

### 2.3. Further Processing Processes

Subsequent processing includes panel pressing, flat film and blown film extrusion.

Panels are pressed using a laboratory press (LP-S-20, Labtech Engineering Company Ltd., Samutprakarn, Thailand). Firstly, the pellets are preheated at 180 °C for 2 min. After venting (5 s) full pressing is applied at 5 MPa, 180 °C for 3 min. Then the cooling cycle follows (2 min). The pressed panels have a dimension of approximately 0.8 × 150 × 150 mm^3^.

Flat films are fabricated using a single-screw extruder (LE 25-30/C, diameter: 25 mm, L/D ratio: 30, LabTech Engineering company Ltd., Samutprakarn, Thailand) in combination with a chill roll attachment (LCR-300, slot die gap: 0.8 mm, maximum die width: 300 mm, LabTech Engineering company Ltd., Samutprakarn, Thailand). The screw speed is set to 72 rpm. The temperature range from the heating zones to the die is set to 160–175 °C. The chill roll speed and the pulling-off speed are set to 4.5 and 4.3 m/min, respectively.

To produce blown films, a single-screw extruder (of the same type as that for flat films) is utilized in combination with a blown film plant (LF-400, die gap: 1 mm, die diameter: 50 mm, LabTech Engineering company Ltd., Samutprakarn, Thailand). In this process, PBAT/PLA blend compounds are filled into a hopper of the extruder. The screw speed determining the throughput is set to 45 rpm. The heating zones and the die are set to 170 °C. After pumping the melt through an annular die, the melt is blown with injecting air through the center of the die mandrel. While the flow of supplied air controls the CD, the draw-down speed (set to 4.5 m/min) and the winding speed (set to 3.5 m/min) influence the MD of the blown films.

### 2.4. Characterization

The thermal properties are determined using a differential scanning calorimeter (DSC 204 F1 Phoenix, NETZSCH-Geraetebau GmbH, Selb, Germany). The DSC cell is constantly purged with nitrogen at a flow rate of 10 mL/min. Each PBAT/PLA blend sample (approximately 10 mg) is sealed in an aluminum pan. The temperature program is set as follows: first, the sample is cooled from room temperature to −60 °C with a cooling rate of 10 °C/min. After holding this temperature for 3 min, the sample is heated to 190 °C (heating rate of 10 °C/min). After keeping at 190 °C for 3 min, the second cooling step is performed from 190 to −60 °C (cooling rate of 10 °C/min). This temperature maintains at −60 °C for 3 min, then the second heating step to 190 °C is carried out at the same heating rate.

The morphological properties are examined using a scanning electron microscope (SEM) (Vega3, TESCAN ORSAY HOLDING a.s., Brno, Czech Republic) with SE and BSE detectors. Pressed panels and flat films made of PBAT/PLA blends are fractured under the cryogenic condition in liquid nitrogen. The flat films are cryogenically fractured in both cross and machine directions. The fractured surfaces are sputter-coated with gold for 120 s before observation.

The rheological properties are analyzed using a rheometer (MCR 302, Anton Paar GmbH, Graz, Austria) with a parallel-plate measuring system. The diameter of the upper and lower plates is 25 mm, respectively. The test specimens are stamped out of panels (diameter: 25 mm, thickness: 0.8 mm) and then pre-dried before use. The programming and recording are accomplished using the software RheoCompass provided by the manufacturer. Amplitude sweeps are performed with a shear deformation 0.01–100% (160 °C/10 Hz) to determine the linear viscoelastic region of each sample. Frequency sweeps are carried out at a strain of 1% and a frequency range of 0.1–10 Hz, at 160 °C. After reaching 160 °C with a tolerance of 0.1%, each sample is heated for 2 min.

The tensile properties are examined using a material testing machine with a 500 N load cell (BT2-EXMACRO.ETH.011, ZwickRoell GmbH & Co. KG, Ulm, Germany). According to DIN EN ISO 527-3: 2019, standard specimen type 2 is used for pressed panels and blown films, while type 5 is applied for flat films. The film specimens are tested in both CD and MD due to the anisotropy. Before testing, all test specimens are conditioned at 23 °C and 50% relative humidity for at least 24 h. Flat films are tested using a universal tester (5567A, Instron GmbH, Darmstadt, Germany) with a 2 kN load cell and pneumatic grips. A crosshead speed of 1 mm/min is set to determine the tensile modulus. Subsequently, an increased cross speed (50 mm/min for flat films; 200 mm/min for panels and blown films) is set to measure the tensile strength and elongation at break.

The tear propagation resistance of blown films is investigated by two methods: trouser tear test according to DIN EN ISO 6383-1: 2016 and Elmendorf tear test according to DIN EN ISO 6383-2: 2004. The trouser tear test applies a constant speed of 200 mm/min utilizing a tensile testing machine with a 500 N load cell in combination with a software (machine: BT2-EXMACRO.ETH.011, software: TestXpertIII, ZwickRoell GmbH & Co. KG, Ulm, Germany) in CD and MD. However, the Elmendorf tear test applies a high speed of tearing through a pendulum (Electronic Elmendorf ProTear, Thwing-Albert Instrument Company, West Berlin, NJ, USA) in both test directions.

## 3. Results and Discussion

### 3.1. Manufacturability of Pressed Panels, Flat Films and Blown Films

Test specimens made of PBAT/PLA blends with different thicknesses are processed using three various methods (Table 1).

Optical homogeneous panels are successfully pressed with a thickness of 800 µm.

Flat films with a thickness of approximately 110 µm and a width of around 130 mm are producible with most blends. However, it fails when the PBAT/PLA blend ratio is 50/50 or 40/60.

Blown films (thickness: 25 µm; lay-flat (LF) width: 180 mm) are produced, when PBAT has a weight percentage of at least 60%. To control the bubble-forming process, several parameters have to be set. The relevant processing parameters (Table 2) include the blow-up ratio (BUR), draw-down ratio (DDR) and the forming ratio (FR) [21]. The FR influences the molecular orientation, meanwhile, describes the balance of stretching between the cross and machine direction [21]. 

**Figure 1:**
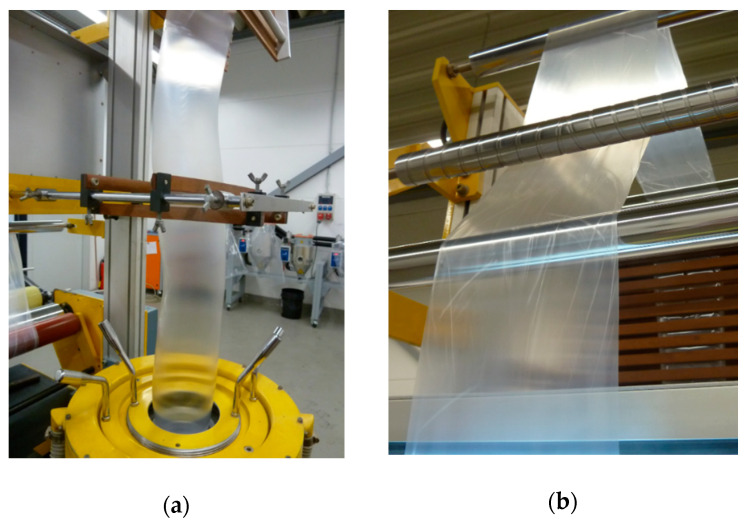
Observation of the instability of the blown film production process: (**a**) helical instability; (**b**) blown films with wrinkles.

With a PLA content of 10–30 wt.%, the thickness of blown films varies slightly. When the PLA content increases to 40% by weight, the production process begins to become unstable. A further increase of the PLA content leads to wrinkles of blown films increasingly (Figure 1); furthermore, the mentioned thickness and width are not reachable any more using the existing equipment.

To understand the origin of the processability of PBAT/PLA blends, the blend miscibility, thermal properties, rheology and morphology are investigated in the following subchapter.

### 3.2. Miscibility Prediction

A common method to predict the miscibility is to use solubility parameters (SP). Hildebrand SP of PBAT is 21.9 and the one of PLA is 20.7 or 19.9 [MPa^1/2^] respectively (Table 3).

Due to the small difference of the solubility parameters, the PBAT/PLA blends need be experimentally investigated, whether they are practically miscible, partially miscible or immiscible.

In this paper, miscibility is a thermodynamic term describing the behavior of a polymer blend by specifying the number of phases, while compatibility is a technical term defining the blend property profile from the practical perspective of a certain application [7]. Despite the subtle difference, miscibility and compatibility are not completely independent terms. A partially miscible or immiscible polymer blend can also be compatible.

### 3.3. Thermal Properties and Miscibility

Glass transition temperatures (*T_g_*) of a binary polymer blend can reveal whether two components are miscible [24]. The full miscibility of a polymer blend is characterized by a single *T_g_*; In a partially miscible system, there are two *T_g_* values that depend on the composition; Immiscible polymers show two *T_g_* values for pure components without changes at different compositions.

**Figure 2:**
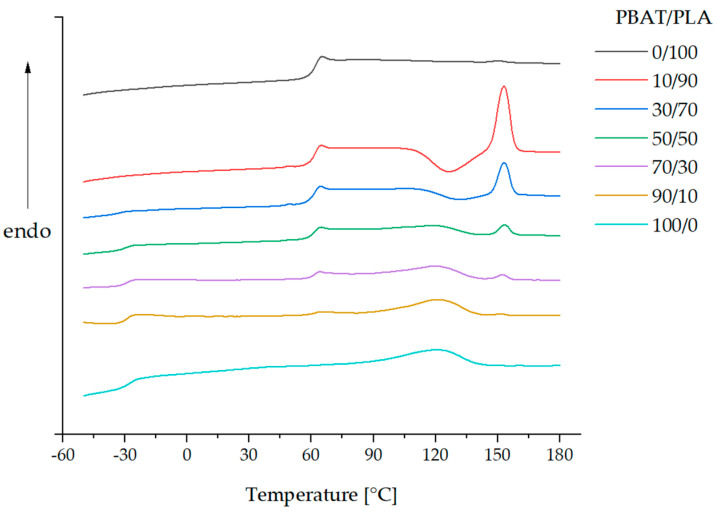
DSC thermograms: the second heating curves of PBAT/PLA blends.

DSC thermograms (Figure 2) display the second heating curves of pure PBAT, pure PLA and PBAT/PLA blends with different compositions.

The first heating curve removes the previous thermal history of samples. The second heating scan of the pure PBAT indicates a *T_g_* at −28.3 °C and a *T_m_* of 120.4 °C (Table 4) The melting peak of PLA (0/100) is very weak, but it is detectable by the analytical software (Figure S1). The reason is probably the slow crystallization rate of neat PLA. The *T_g_* and *T_m_* of neat PLA are 61.6 and 149.6 °C, respectively. The PBAT/PLA blends generally represent two separated almost unchanged glass transition temperatures (*T_g_* ~ −30 °C and 61 °C) and two melting temperatures (*T_m_* ~ 120 °C/152 °C) corresponding to those for PBAT and PLA, which is in agreement with the observation of the blends in the literature [12,13]. Moreover, the melting region of PBAT and the cold crystallization of PLA occur in a similar temperature range. Due to the overlap of the energetically opposite processes in the same temperature range, it is inappropriate to calculate the degree of crystallinity of PLA in the blends.

If PBAT and PLA were miscible at the molecular level, then there should be a shift in the glass transition temperatures according to the Fox Equation (1) [24]:

(1) $$\frac{1}{T_{g}}\;=\;\frac{x\left(\mathrm{PBAT}\right)}{T_{g}\left(\mathrm{PBAT}\right)}+\frac{x\left(\mathrm{PLA}\right)}{T_{g}\left(\mathrm{PLA}\right)}$$

A miscible PBAT/PLA (10/90) blend would have a single *T_g_* at 42.2 °C according to the calculation using the Fox equation. However, the detected single *T_g_* of the blend (61.4 °C) corresponds to the *T_g_* values of the pure PLA (61.6 °C). The almost unchanged *T_g_* implies that PLA exhibits no improvement in the chain mobility by adding PBAT. The appearance of a single *T_g_* is probably due to the low PBAT content (10%) in the blend and the low cooling rate (10 °C/min). Among all PBAT/PLA blends, the *T_g_* difference varies from 89.7 and 93.2 °C. The ∆*T_g_* difference between blends is not higher than 3.5 °C, which agrees with the previous research results [8,11]. Therefore, the DSC results point to thermodynamically limited miscibility of PBAT/PLA blends. Furthermore, the PBAT-rich blends are slightly more miscible than PLA-rich blends. 

### 3.4. Rheological Properties and Miscibility

**Figure 3:**
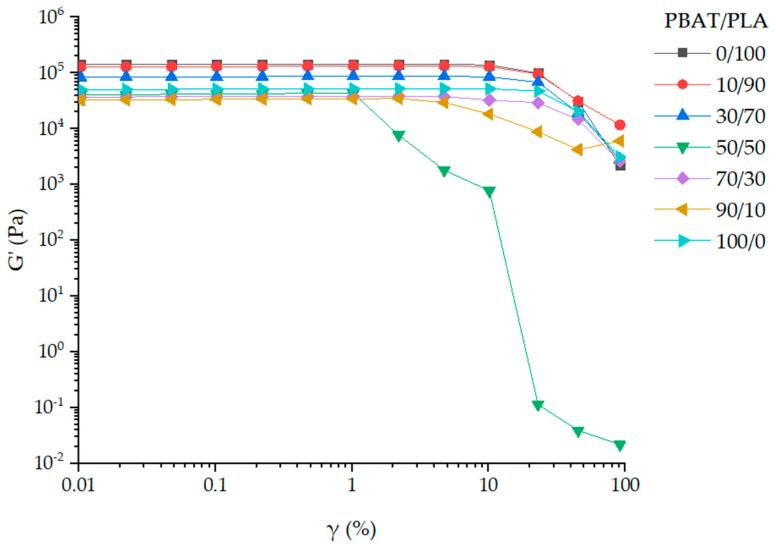
Amplitude sweep tests of PBAT/PLA melts, neat PBAT and PLA as control samples at 160 °C.

To determine the limit of the linear viscoelastic (LVE) range, amplitude sweeps are performed at a frequency of 62.8 rad/s and a temperature of 160 °C (Figure 3).

At 160 °C, the storage modulus (*G’*) of the neat PLA melt (0/100) shows the highest storage modulus of all samples at lower deformations. With increasing content of PBAT (from 0 to 90 wt.%), *G’* decreases at lower deformations, which contradicts the observation of Gu et al. [25] with a PBAT content of 0–30 wt.% at 180 °C. The *G’* of pure PBAT (100/0) is slightly higher than the *G’* of PBAT/PLA blends with low PLA content (10 and 30%). The limit of the LVE range of pure polymers and all blends is greater than 1%. The sharp decrease of G’ in PBAT/PLA (50/50) with a deformation from 1 to 100% indicates a phase inversion. Therefore, the linear viscoelastic properties of PBAT/PLA melts are conducted at a strain of 1%.

**Figure 4:**
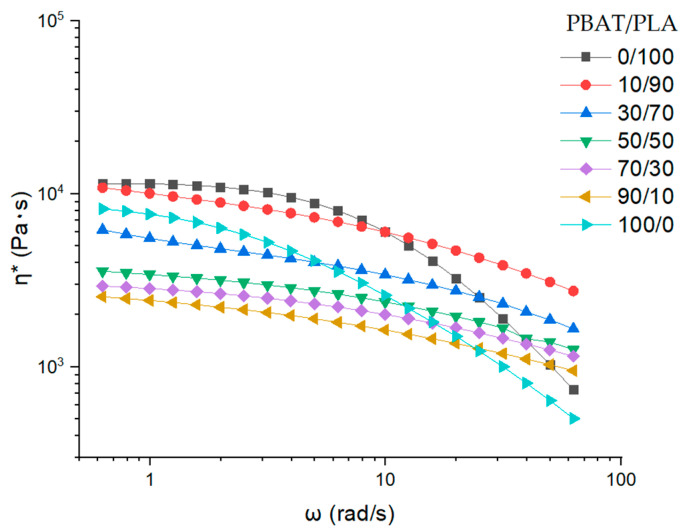
Angular frequency dependence of complex viscosity η*.

The complex viscosity (η*) decreases with the angular frequency ranging from 0.628 to 62.8 rad/s at 160 °C (Figure 4), revealing that the PBAT/PLA blends and the pure polymers (PBAT and PLA) present shear-thinning behavior. At lower frequencies, the complex viscosity of the samples increases with increasing PLA content (from 10 to 100 wt.%), which is contradictory to the result of Li et al. at 190 °C [26]. The increase of the viscosity is probably contributed by relatively high molecular weight PLA, which allows forming more entanglements. However, pure PBAT (100/0) shows even higher complex viscosity than PBAT/PLA blends with a PLA content of less than 70 wt.% at lower frequencies. However, the complex viscosity of pure PBAT decreases more dramatically with the increasing frequency. The reason for this is maybe the increasing loss of physical entanglements at higher frequencies at 160 °C.

**Figure 5:**
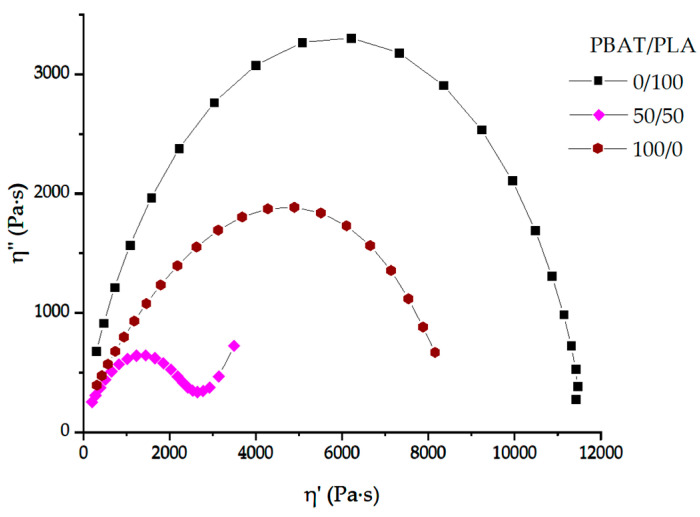
Cole–Cole plot (imaginary viscosity versus real viscosity) for PBAT/PLA (50/50) blend, pure PBAT and PLA at 160 °C.

Pure PLA, PBAT and PBAT/PLA (50/50) blend are characterized at 160 °C using the Cole–Cole plot (Figure 5). Both pure polymers show only one circular arc in the curve. However, for the uncompatibilized PBAT/PLA (50/50) blend, the Cole–Cole-Plot yields an S-shape. The left side shows the relaxation of one blend component (polymer matrix) and the right side is attributed to another blend component (droplet relaxation) [26,27,28]. The two different relaxation mechanisms correspond to two phases. Furthermore, the appearance of the tail on the right side implies a phase inversion from a droplet-matrix morphology to a co-continuous morphology in the internal structure [29]. 

### 3.5. Morphological Properties and Miscibility

The morphology including the size and size distribution of the minor phase influences the mechanical properties of a blend [30]. In this subchapter, the morphology of PBAT/PLA blends of both isotropic pressed panels and anisotropic flat films is presented.

**Figure 6:**
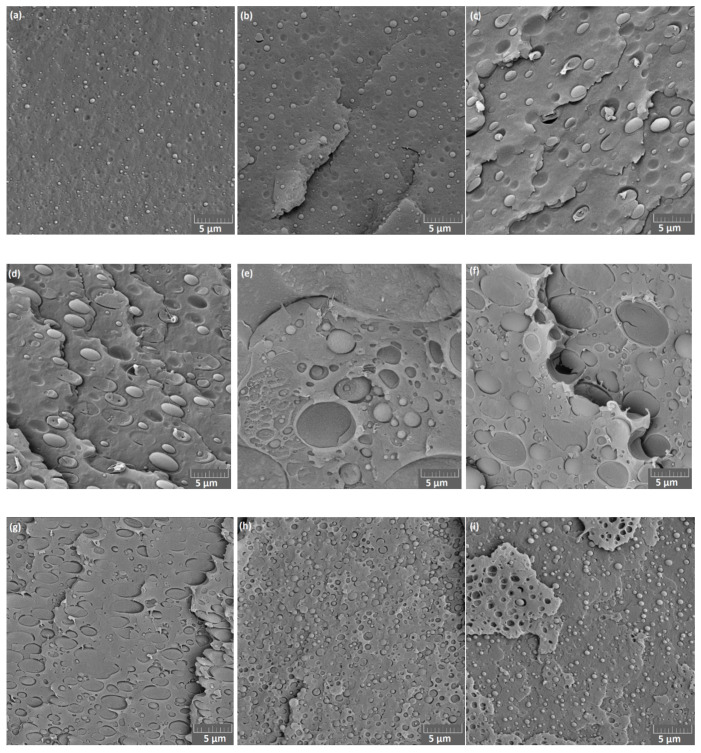
SEM of cryofractured surface of pressed panels (800 µm) made of PBAT/PLA blends with various weight ratios: (**a**) 90/10, (**b**) 80/20, (**c**) 70/30, (**d**) 60/40, (**e**) 50/50, (**f**) 40/60, (**g**) 30/70, (**h**) 20/80, (**i**) 10/90.

SEM micrographs (Figure 6) show cryogenically fractured surfaces of panels made of PBAT/PLA blends with various ratios. The PBAT/PLA (90/10) blend presents a smooth surface with small spherical PLA droplets of about 1 µm. Increasing PLA from 10 to 30 wt.%, the diameter of droplets grows, and the fractured surface becomes rough gradually. In blends with 40–60 wt.% PLA, the fractured surface is rough and partially stratified with large platelet- or column-shaped phase separation (diameter up to 8 µm). Moreover, it is noticeable that a few small droplets of one phase are immersed in the large droplets of the other phase, which exhibits two-phase characteristics. With increasing PLA content from 70 to 90 wt.%, the size of embedded PBAT droplets decreases in the matrix of PLA.

In some cases, voids are noticeable at the interface, indicating low interfacial adhesion between the PBAT and PLA phases. It seems that the fracture went preferentially through the interface between spherical droplets and matrix. The typical sea-islands morphology in SEM depicts poor miscibility between PBAT and PLA, which is consistent with the observation in other studies [9,13].

**Figure 7:**
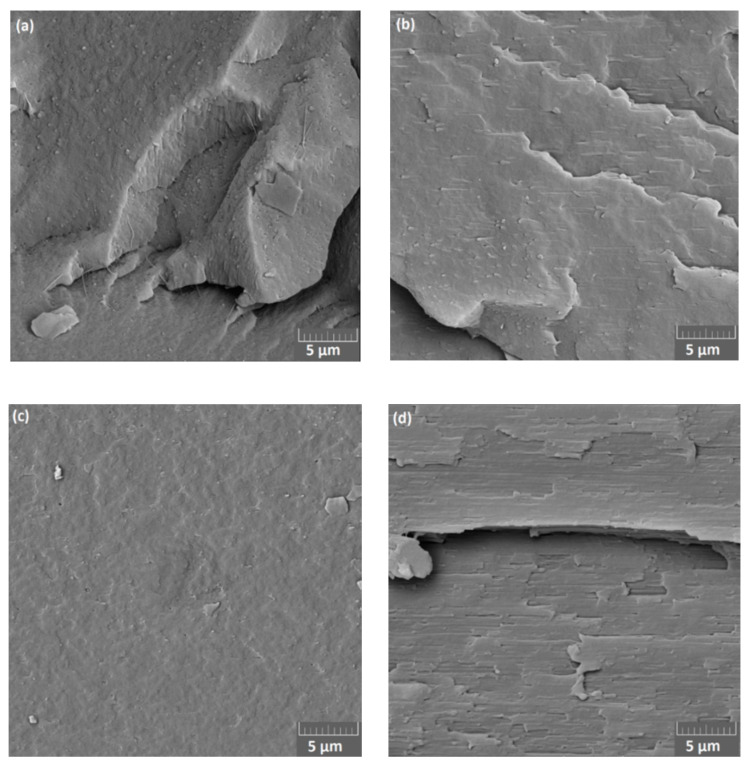
SEM of cryofractured surfaces of flat films made of PBAT/PLA blends: (**a**) 90/10 in CD, (**b**) 90/10 in MD, (**c**) 60/40 in CD, (**d**) 60/40 in MD.

SEM micrographs (Figure 7) demonstrate the cryogenically fractured surfaces of PBAT/PLA (90/10 and 60/40) blend flat films in CD and MD. The morphology of the flat films differs greatly in the directions. In CD, small spherical PLA droplets disperse in the PBAT matrix. In MD, PBAT/PLA (90/10) blend presents an elongated ribbon-like PLA phase in the PBAT matrix towards the melt stretching direction (MD), probably due to the orientation of the polymer chains. PBAT/PLA (60/40) exhibits in MD stratified phases and PBAT is the continuous phase, which is similar to the observation reported by Arruda et al. [12]. All flat film samples exhibit phase separation behavior (further SEM micrographs in Figure S2), indicating poor miscibility of flat films made of PBAT/PLA blends.

### 3.6. Tensile Properties

To determine the influence of the PBAT/PLA ratio on the tensile properties, the pressed panels, flat films and blown films with different ratios are tested, respectively. The Origin’s normality test confirms that data used in the evaluation are significantly drawn from a normally distributed population at the 0.05 level. The mean values of samples are compared using an ANOVA one-way test (Tukey) to determine significant differences.

**Figure 8:**
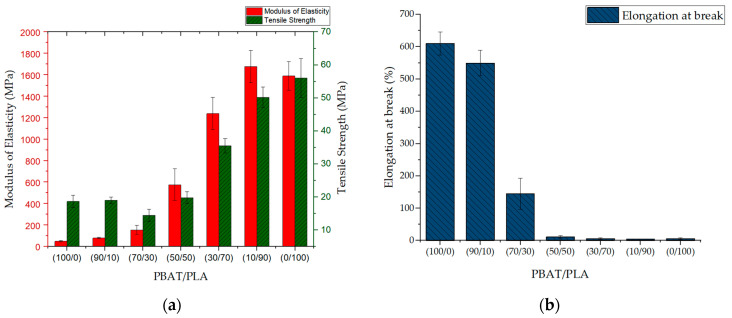
Tensile properties of pressed panels made of PBAT/PLA blends, pure PBAT and PLA. (**a**) modulus of elasticity and tensile strength, (**b**) elongation at break.

Due to the isotropy, test specimens obtained from pressed panels do not differ in different directions. The PBAT/PLA (100/0), (90/10) and (50/50) panel samples do not show significant differences in the tensile strength (Figure 8a). Compared with these three samples, the (70/30) blend panel presents lower tensile strength, which matches the results of Deng et al. [8]. This decrease in tensile strength is probably due to the morphological change: the average droplet diameter of the blends increases distinctly when the PLA content increases from 10 to 30 wt.% (Figure 6). With the increase of PLA content (50–90 wt.%), both the modulus of elasticity and the tensile strength tend to rise. The PBAT/PLA (10/90) blend panel is not significantly different from the neat PLA (0/100) panel regarding the modulus of elasticity and tensile strength, respectively. On the other hand, the elongation at break shows the opposite tendency (Figure 8b), i.e., with the increasing PLA content, this value drops from approximately 600% (pure PBAT) to less than 6% (PBAT/PLA (30/70), (10/90) blends and neat PLA).

Flat films and blown films have anisotropy due to the orientation of the polymer chains during processing. Therefore, the film samples are tested both in the CD and MD.

The modulus of elasticity (MOE) is predictable using the rules of mixing. Parallel Model (2) and Series Model (3) predict the upper and lower boundaries of blend behavior, respectively.

(2) $$E_{b}\;=\;\varphi_{1}E_{1}+\varphi_{2}E_{2}$$

(3) $$E_{b}\;=\;\frac{E_{1}E_{2}}{\left(\varphi_{1}E_{2}+\varphi_{2}E_{1}\right)}$$

*E*_1_ and *E*_2_ are the modulus of components 1 and 2, respectively, while *E*_b_ is the modulus of the blend. The modulus of elasticity is about 88 MPa for PBAT [31] and 3500 MPa for PLA [7]. Both polymers have almost the same density (1.25 kg/m^3^). $\varphi_{1}$ and $\varphi_{2}$ are the corresponding volume fractions. The experimental MOE of all blends is between the limits of the Parallel and Series Models (Table 5). Taking the standard deviation into account, the MOE values of the MD are relatively close to the Parallel Model, indicating strong interactions between the two components. Therefore, PBAT and PLA show some compatibility in the MD of their flat films, although they have shown poor miscibility in DSC and SEM.

**Figure 9:**
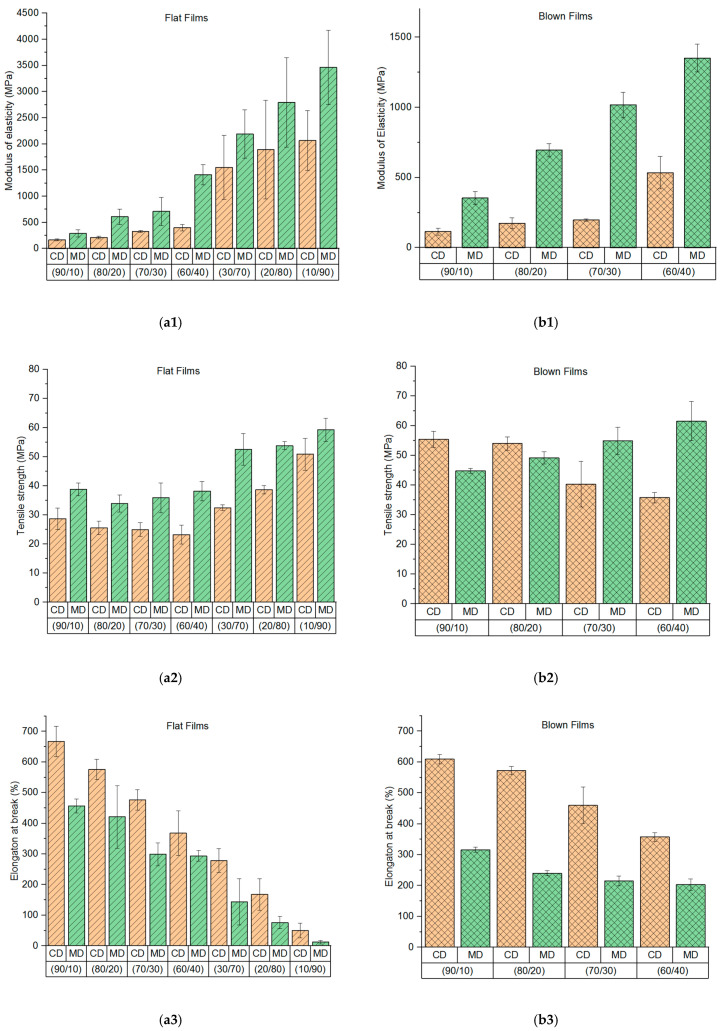
Tensile properties of flat films and blown films made of PBAT/PLA blends in cross direction (CD) and machine direction (MD): (**a1**) modulus of elasticity of flat films, (**b1**) modulus of elasticity of blown films, (**a2**) tensile strength of flat films, (**b2**) tensile strength of blown films, (**a3**) elongation at break of flat films, (**b3**) elongation at break of blown films.

The modulus of elasticity tends to increase in both directions with increasing PLA weight percentage in flat films (Figure 9(a1)) as well as in blown films (Figure 9(b1)). That indicates that PLA contributes to enhancing the modulus of elasticity.

The tensile strength of flat films is generally lower in CD than in MD (Figure 9(a2)). This phenomenon is probably due to the melt stretching only in one direction (MD). Therefore, molecules align themselves in this direction. According to the statistical analysis, the PBAT/PLA (90/10), (80/20), (70/30) and (60/40) flat films are not significantly different regarding the tensile strength in both directions. The tensile strength of PBAT/PLA (30/70), (20/80) and (10/90) flat films tends to increase. However, these samples do not show significant differences in the MD. The tensile strength of PBAT/PLA (10/90) flat film is significantly higher than the one of (80/20) and (70/30) flat films.

Compared with the uniaxial stretched flat films, blown films are drawn biaxially. The tensile strength of blown films tends to decrease in CD but increase in MD (Figure 9(b2)) with increasing PLA content, when the parameter settings of the blown film production remain constant.

The elongation at break tends to decrease with the increasing PLA content. This phenomenon occurs in both flat films (Figure 9(a3)) and blown films (Figure 9(b3)) in both directions when the PLA content increases from 10 to 40 wt. %.

In general, PLA contributes to enhancing the modulus of elasticity in films. When the PLA content is 10–40 wt.%, it has a minor influence on the tensile strength of films. However, the increasing PLA content effectively reduces the elongation at break of the films. Both flat films and blown films have higher tensile strength than pressed panels with the same composition, probably due to the processing of blend compounds into test specimens. During film production, the blends are compounded again with an extruder, which results in better-homogenized materials than pressed panels. The blown films, flat films and pressed panels have different thicknesses (25, 110 and 800 µm, respectively). The thicker the samples, the greater the probability of defects in the sample.

### 3.7. Tear Propagation Resistance of Blown Films

In addition to the tensile properties, tear resistance also plays an important role in the mechanical properties of a blown film [32]. The tear resistance includes tear initiation and tear propagation resistance. The resistance to tear propagation is expected to be much lower than the resistance of tear initiation [33]. Moreover, not only the sufficient tear propagation resistance of blown films is required, but also a minimum possible film thickness is necessary to achieve competitive material costs and enhanced biodegradability. To our best knowledge, the tear propagation resistance of PBAT/PLA blend films is compared for the first time using two tear propagation test methods.

The blown films are tested according to the direction using the trouser tear method and Elmendorf tear method (Table 6). Both methods show the same tendency that the tear propagation resistance of the blown films decreases with increasing PLA content (10–40 wt.%). The blown film made of PBAT/PLA (90/10) has the highest tear propagation resistance (113.5 ± 2.1 N/mm in CD and 48.9 ± 5.0 N/mm in MD). A good trend correlation is observed between the tear propagation resistance (Table 6) and their elongation at break (Figure 9(b3)) of the blown films.

**Figure 10:**
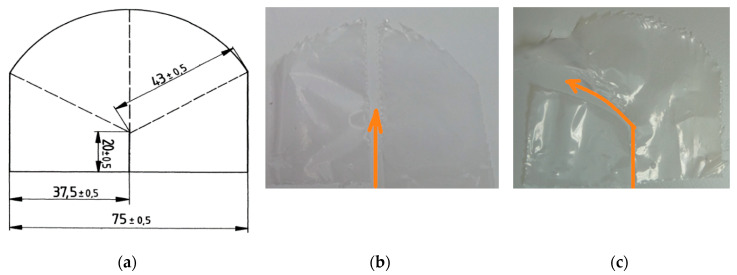
Test Specimen according to the Standard DIN EN ISO 6383-2: 2004 [20] (**a**): Crack course in MD, (**b**): straight crack, (**c**): curved cracks.

By using the trouser tear method, the measurement of all samples in both directions is successful. However, the Elmendorf tear method cannot measure the PBAT/PLA (90/10) blown film sample in MD correctly. Test specimens for the Elmendorf method have a constant-radius testing length (Figure 10). The results depend strongly on the course of crack during the test: straight cracks result in low values; curved cracks cause higher values. Since curved cracks occur almost in all PBAT/PLA (90/10) samples in MD, the results by the Elmendorf method are not valid for evaluating this sample. Therefore, the trouser tear method is more applicable to differentiate highly extensible blown films than the Elmendorf tear method.

## 4. Conclusions

PBAT and PLA were melt-blended in a wide range of ratios and processed into pressed panels, flat films and blown films. The manufacturability, miscibility and mechanical properties of uncompatibilized PBAT/PLA blends are examined.

Optical homogenous panels (thickness: 0.8 µm) were pressed with all blend ratios. Flat film production (thickness: 110 µm, width: 130 mm) failed when the PBAT/PLA blend ratio is 50/50 or 40/60. The reason is probably the morphological change from droplet-matrix to co-continuous phases in internal structure, which is confirmed in the rheological test. Blown films (thickness: 25 µm, lay-flat width: 180 mm) were successfully fabricated with a PLA content up to 40 wt.%. The processing parameters were set to BUR = 2.3, DDR = 40 and FR = 17.5.

Hildebrand solubility parameters of PBAT and PLA are close, predicting theoretically good miscibility between them. However, the DSC analysis represents two separated almost unchanged *T_g_* in PBAT/PLA blends corresponding to those for PBAT and PLA. The measured values are inconsistent with the values calculated using the Fox equation, indicating limited miscibility between PBAT and PLA. Among the blends, PBAT-rich blends are slightly more miscible than PLA-rich blends. The rheological test shows that all PBAT/PLA blends have shear-thinning behavior. The Cole–Cole-Plot shows two different relaxation mechanisms correspond to two phases in PBAT/PLA (50/50) and a phase inversion from a droplet-matrix morphology to a co-continuous morphology in the internal structure. SEM presents two-phase characteristics of PBAT/PLA blends and anisotropic morphologies of flat films caused by melting stretching in the processing process.

Tensile tests reveal that PLA generally contributes to the modulus of elasticity and tensile strength of PBAT/PLA blends, but the elongation at break decreases with increasing PLA content. Taking the standard deviation into account, the modulus of elasticity of flat films in MD fits the upper limit of the blend behavior, indicating some compatibility despite limited miscibility. With the same parameter settings in blown film production and increasing PLA content (10–40 wt.%), the tensile strength of blown films tends to decrease in CD but increase in MD while the elongation at break increases. The tear propagation resistance also increases with the increasing PBAT content. A good correlation exists between the tear propagation resistance and the elongation at break of the blown films. Furthermore, the trouser tear method is more applicable to differentiate highly extensible blown films than the Elmendorf tear method.

Improved mechanical properties especially the tear propagation resistance are required for film applications of PBAT/PLA blends. This is achievable by blend compatibilization together with the optimization of processing parameters. Meanwhile, biodegradability should not be sacrificed. The parameter settings are important for films to reach a balance of the mechanical properties in different directions. Additionally, to address the PBAT/PLA blends into the market, the material costs should be taken into account, which is partly realizable by the minimum possible film thickness.

## Figures and Tables

**Table 1:** Manufactured specimens made of PBAT/PLA blends with various ratios.

Specimen	Thickness [µm]	90/10	80/20	70/30	60/40	50/50	40/60	30/70	20/80	10/90
Panel	800	✓	✓	✓	✓	✓	✓	✓	✓	✓
Flat film	110	✓	✓	✓	✓			✓	✓	✓
Blown film	25	✓	✓	✓	✓					

**Table 2:** Processing parameters of the blown film production and the equations.

Parameter	Value	Equation
BUR	2.3	$\mathrm{BUR}\;=\frac{D_{b}}{D_{d}}$
DDR	40.0	$\mathrm{DDR}\times \mathrm{BUR}\;=\frac{\varepsilon_{0}}{\varepsilon_{f}}$
FR	17.5	$\mathrm{FR}\;=\frac{\mathrm{DDR}}{\mathrm{BUR}}$

Legend: *D_b_* is the bubble diameter (114.6 mm) calculated from the lay-flat width: ($D_{b}\;=\;2\times \frac{LF}{\pi }$); *D_d_* is the die diameter (50 mm); *ε_0_* is the die gap (1000 µm) and *ε_f_* is the film thickness (25 µm).

**Table 3:** Hildebrand solubility parameters of PBAT and PLA.

Polymer	Hildebrand SP [MPa^1/2^]	Ref
PBAT	21.9	[22]
PLA	20.7	[22]
	19.9	[23]

**Table 4:** Thermal characteristics from the second heating curves of pure PBAT, PLA and PBAT/PLA blends.

PBAT/PLA Weight Ratio	*T_g_* (PBAT) [°C]	*T_m_* (PBAT) [°C]	*T_g_* (PLA) [°C]	*T_m_* (PLA) [°C]
0/100	-	-	61.6	149.6
10/90	**	*	61.4	153.1
20/80	**	*	61.2	153.4
30/70	−32.1	*	60.8	153.3
40/60	−32.0	*	61.2	153.1
50/50	−30.0	119.6	61.1	153.4
60/40	−29.8	119.3	60.9	152.6
70/30	−28.8	120.3	60.9	152.1
80/20	−29.0	120.3	61.5	151.8
90/10	−29.3	120.9	61.0	151.8
100/0	−28.3	120.4	-	-

Legend: -: not applicable. *: no specification due to partial overlap of the *T_m_* (PBAT) and cold-crystallization region of PLA. **: *T_g_* is not resolved.

**Table 5:** Experimental and theoretical modulus of elasticity of flat films made of PBAT/PLA blends.

Flat Film of PBAT/PLA	Exptl. MOE (CD) [MPa]	Exptl. MOE (MD) [MPa]	Theo. (Parallel) MOE [MPa]	Theo. (Series) MOE [MPa]
90/10	166 ± 20	290 ± 68	429	98
80/20	210 ± 26	607 ± 143	770	109
70/30	329 ± 20	710 ± 269	1112	124
60/40	397 ± 63	1410 ± 193	1453	144
30/70	1551 ± 610	2190 ± 461	2476	277
20/80	1889 ± 943	2794 ± 853	2818	400
10/90	2065 ± 573	3460 ± 709	3159	718

**Table 6:** Tear Propagation Resistance of Blown Films in Cross and Machine Direction.

PBAT/PLA	Trouser Tear CD [N/mm]	Trouser Tear MD [N/mm]	Elmendorf CD [N/mm]	Elmendorf MD [N/mm]
90/10	113.5 ± 2.1	48.9 ± 5.0	50.6 ± 0.9	*
80/20	33.2 ± 1.2	19.2 ± 2.0	48.5 ± 1.1	45.6 ± 2.1
70/30	16.6 ± 0.8	8.0 ± 0.7	27.6 ± 0.8	11.5 ± 0.6
60/40	12.9 ± 0.5	5.9 ± 0.3	8.8 ± 0.5	5.9 ± 0.3

Legend: *: Result is not valid according to the standard DIN EN ISO 6383-2: 2004.

## Supplementary Materials

The following are available online at https://www.mdpi.com/1996-1944/13/21/4897/s1, Figure S1: DSC of pure PLA analyzed by the software Netzsch Proteus Thermal analysis, Figure S2: SEM of further flat films made of PBAT/PLA blends: (a) (80/20) in CD, (b) (80/20) in MD, (c) (70/30) in CD, (d) (70/30) in MD, (e) (20/80) in CD, (f) (20/80) in MD, (g) (10/90) in CD, (h) (10/90) in MD.
