# Supplementary material for: Uncompatibilized PBAT/PLA Blends: Manufacturability, Miscibility and Properties
Source: Materials (Basel). 2020 Oct 31;13(21):4897. doi: 10.3390/ma13214897 (PMC7662590; doi:10.3390/ma13214897)
Supplement: Supplementary file 1 [file materials-13-04897-s001.pdf]

materials MDPI

Supplementary Materials

Uncompatibilized PBAT/PLA Blends: Manufacturability, Miscibility and Properties

Shen Su [1,2,*], Mona Duhme [1] and Rodion Kopitzky [1]

[1] Department of Circular and Bio-based Plastics, Fraunhofer UMSICHT, Institute for Environment, Safety and Energy Technology, Osterfelder Str. 3, 46047 Oberhausen, Germany; mona.duhme@umsicht.fraunhofer.de (M.D.); rodion.kopitzky@umsicht.fraunhofer.de (R.K.)

[2] Department of Mechanical Engineering, Ruhr-University Bochum, Universitaetsstr. 150, 44780 Bochum, Germany

* Correspondence: shen.su@umsicht.fraunhofer.de; Tel. +49-208-85981422

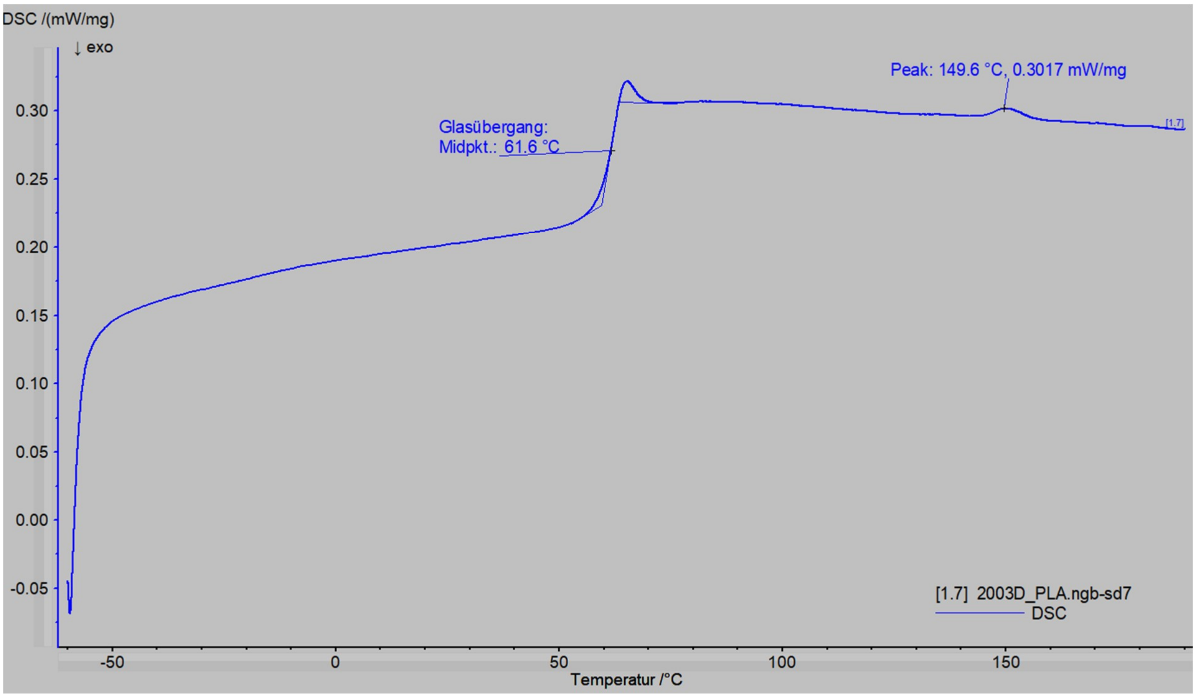

Figure S1. DSC of pure PLA analyzed by the software Netzsch Proteus Thermal analysis

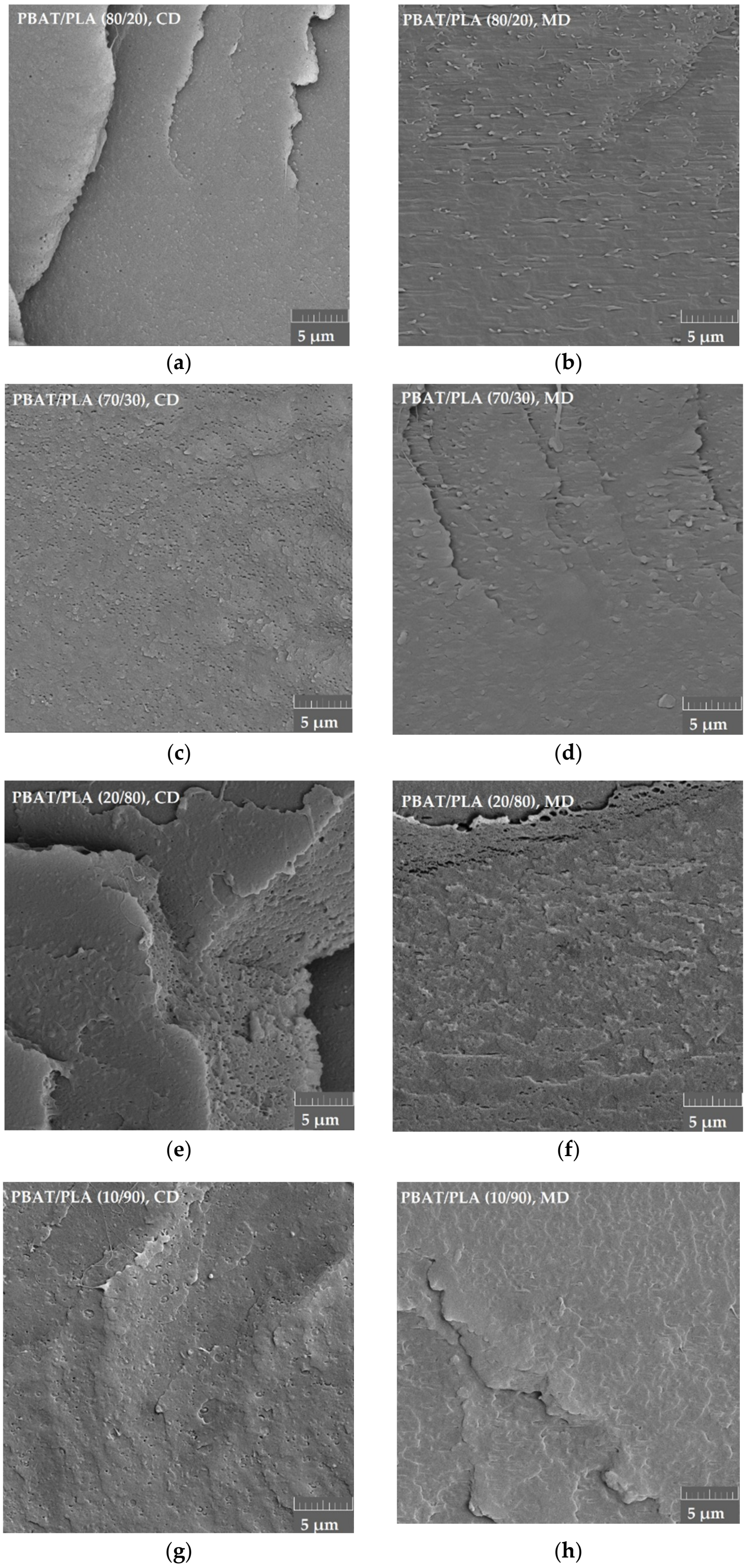

Figure S2. SEM of further flat films made of PBAT/PLA blends: (**a**) (80/20) in CD, (**b**) (80/20) in MD, (**c**) (70/30) in CD, (**d**) (70/30) in MD, (**e**) (20/80) in CD, (**f**) (20/80) in MD, (**g**) (10/90) in CD, (**h**) (10/90) in MD.
